# Financial Assistance for Health Security: Effects of International Financial Assistance on Capacities for Preventing, Detecting, and Responding to Public Health Emergencies

**DOI:** 10.34172/ijhpm.2021.120

**Published:** 2021-09-01

**Authors:** Matthew R. Boyce, Mark J. Meyer, John D. Kraemer, Rebecca Katz

**Affiliations:** ^1^Center for Global Health Science & Security, Georgetown University Medical Center, Georgetown University, Washington, DC, USA.; ^2^Department of Mathematics and Statistics, Georgetown University, Washington, DC, USA.; ^3^Department of Health Systems Administration, Georgetown University, Washington, DC, USA.

**Keywords:** Capacity Development, Financing, Global Health, Health Policy, Health Security, Health System Strengthening

## Abstract

**Background:** Health security funding is intended to improve capacities for preventing, detecting, and responding to public health emergencies. Recent years have witnessed substantial increases in the amounts of donor financial assistance to health security from countries, philanthropies, and other development partners. To date, no work has examined the effects of assistance on health security capacity development over time. This paper presents an analysis of the time-lagged effects of assistance for health security (AHS) on levels of capacity.

**Methods:** We collected publicly available health security assessment scores published between 2010 and 2019 and data relating to financial AHS. Using validated methods, we rescaled assessment scores on analogous scales to enable comparison and binned them in quartiles. We then used a distributed lag model (DLM) in a Bayesian ordinal regression framework to assess the effects of AHS on capacity development over time.

**Results:** Strong evidence exists for associations between financial assistance and select capacities on a variety of lagged time intervals. Financial assistance had positive effects on zoonotic disease capacities in the year it was disbursed, and positive effects on legislation, laboratory, workforce, and risk communication capacities one year after disbursal. Financial assistance had negative effects on laboratory and emergency response capacities two years after it was disbursed. Financial assistance did not have measurable effects on coordination, antimicrobial resistance (AMR), food safety, biosafety, surveillance, or response preparedness capacities over the timeframe considered.

**Conclusion:** Financial AHS is associated with positive effects for several core health security capacities. However, for the majority of capacities, levels of funding were not significantly associated with capacity level, though we cannot fully exclude endogeneity. Future work should continue to investigate these relationships in different contexts and examine other factors that may contribute to capacity development.

## Background

**Key Messages**

** Implications for policy makers**

- Evidence exists for associations between financial assistance for health security (AHS) and capacity level on a variety of time intervals.
- Strong positive associations were observed between international financial assistance and improvements in zoonotic disease capacities in the year assistance was disbursed; and improvements in legislation, laboratory, workforce, and risk communication capacities one year after the disbursal.
- Negative associations were observed between international financial assistance and laboratory and emergency response capacities two years after assistance was disbursed, but these results should be interpreted with caution.
- International financial assistance did not measurably impact coordination, antimicrobial resistance (AMR), food safety, biosafety, surveillance, or response preparedness capacities on the time interval considered.

** Implications for the public**

There have been substantial increases in the amounts of international financial assistance for health in recent years. This trend is likely to continue in the aftermath of the coronavirus disease 2019 (COVID-19) pandemic as governments work to build public health capacities necessary for strengthening health systems and preventing future infectious disease outbreaks. However, financial assistance does not always translate into the desired impact of improving health or capacity because of complicated pathways and influences from other external, contextual factors. Additionally, the impacts of financial assistance on capacities over time remain unknown. Our assessment seeks to better understand the impacts of financial assistance on capacities for preventing, detecting, and responding to public health emergencies – such as epidemics and pandemics – by analyzing the associations between financial assistance and changes in capacity level over time.

Recent years have witnessed substantial increases in the amounts of donor financial assistance for health (DAH). In this time, the global health community has demonstrated an interest in understanding broad trends in DAH,^1,2^ as well as how DAH has been allocated amongst competing priorities.^3-8^

 Health security is a public health concentration that focuses on preventing, detecting, and responding to public health emergencies. The revised International Health Regulations (IHR) are a legally binding instrument that represent a guiding framework for global health security. These Regulations require Member States to develop and maintain capacities for public health emergency surveillance and response.^9^ With the adoption of the IHR 2005, and the launch of the 2014 Global Health Security Agenda, countries, philanthropies, and other development partners began to explicitly provide assistance for health security (AHS) that was designed to increase national-level health security capacities. Much of this work has been focused on capacity building projects, especially after the 2014 Ebola epidemic exposed the potential consequences of serious deficiencies in public health and healthcare infrastructure.^10^

 In 2010, the World Health Organization (WHO) introduced a self-assessment process as part of the IHR Monitoring and Evaluation Framework for countries to report on IHR implementation, using 256 attributes associated with 8 core capacities and 5 additional hazards and challenges. However, the usefulness of these self-assessments has been questioned owing to criticisms of its generality, reproducibility, granularity.^11^

 After the 2014 Ebola epidemic underscored the shortcomings of public health systems and assessments, the Global Health Security Agenda developed an external assessment tool which was eventually adopted as part of WHO’s revised monitoring and evaluation framework for the IHR.^12^ The IHR Monitoring and Evaluation Framework now endorses a process that uses a Joint External Evaluation (JEE) Tool as one method for assessing national-level health security capacities. The JEE consists of 19 core capacities organized by four main sub-categories – prevent, detect, respond, and points of entry and other IHR-related hazards.^13^ Prevent capacities include national policy and financing, IHR coordination, antimicrobial resistance (AMR), zoonotic disease, food safety, biosafety and biosecurity, and immunization; Detect capacities include laboratory systems, surveillance systems, reporting, and workforce development; Respond capacities include preparedness, emergency response operations, linking public health and security authorities, medical countermeasure and personnel deployment, and risk communication; points of entry and other IHR-related hazard capacities include points of entry, chemical events, and radiation emergencies. These capacities contain a total of 48 indicators that are measured on a 5-step Likert scale using a peer-to-peer model of assessment, in which multidisciplinary teams of external and domestic experts assess health security capacities in a standardized fashion.^12^ This standardized scoring process allows for countries to systematically evaluate their health security capacities, and over 100 countries have completed JEEs.

 In addition to the JEE, countries currently use a combination of other monitoring and evaluation tools for health security, including after-action reports and simulation exercises. The WHO revised the annual self-assessment and JEE tools in 2018 following consultations with countries, and the assessments now closely resemble one another.

 It is widely accepted that ensuring adequate funding for health security initiatives is critical for sustainably developing and maintaining public health capacities. To make the world safer, global institutions, organizations, and countries themselves must follow the assessments with financing, prioritization and management to improve readiness to prevent, detect and respond to disease outbreaks.^14^ There have been multiple calls for a new financing facility for pandemic preparedness, designed to invest not only in global public goods, but also in national level capabilities. This requires an awareness of the current status of funding to identify funding requirements, develop compelling arguments for investment, and prioritize future funding decisions. However, owing to complex causal pathways, financial assistance does not always translate into the desired impact of improved health or capacity.^15^ Further, no work has examined the effects that AHS has had on health security capacities over time. To better understand the impacts of AHS on capacities for preventing, detecting, and responding to public health emergencies, this paper presents an analysis of the effects of AHS and changes in health security capacity over time.

## Methods

###  Data Collection and Sample

 The WHO makes health security assessment scores publicly available. Results for State Parties’ self-assessments for the years 2010-2019 are housed in the Electronic State Party Self-Assessment Annual Reporting Tool (e-SPAR).^16^ JEE scores are published in country mission reports, which are available from the WHO’s JEE mission report website.^17^ We compiled all reported e-SPAR data from 2010-2017 and JEE data into a single Microsoft Excel spreadsheet. These data are publicly available online as a part of the Georgetown Infectious Disease Atlas.^18^ To account for the different scale used from 2010-2017, we used validated methods to rescale JEE and e-SPAR assessment scores so they were on analogous and comparable scales.^11^

 The Global Health Security Tracking Tool is a public, web-based dashboard mapping international financial flows – including both committed and disbursed AHS funding – from development partners to recipient countries.^19^ We compiled data relating to the amounts of AHS committed and disbursed annually between the years 2014 and 2018 and added them to the dataset. If an AHS disbursal spanned multiple years, the total amount disbursed was divided by the number of years (eg, an AHS disbursal listed for 30 million USD from 2015-2017, would be reallocated as three 10 million USD disbursals in each year). Data were excluded if support was an in-kind contribution, if no recipient country was reported, and if the recipient was a region or continental union (eg, Southeast Asia or African Union). We accessed all data in November 2019.

**Figure 1:**
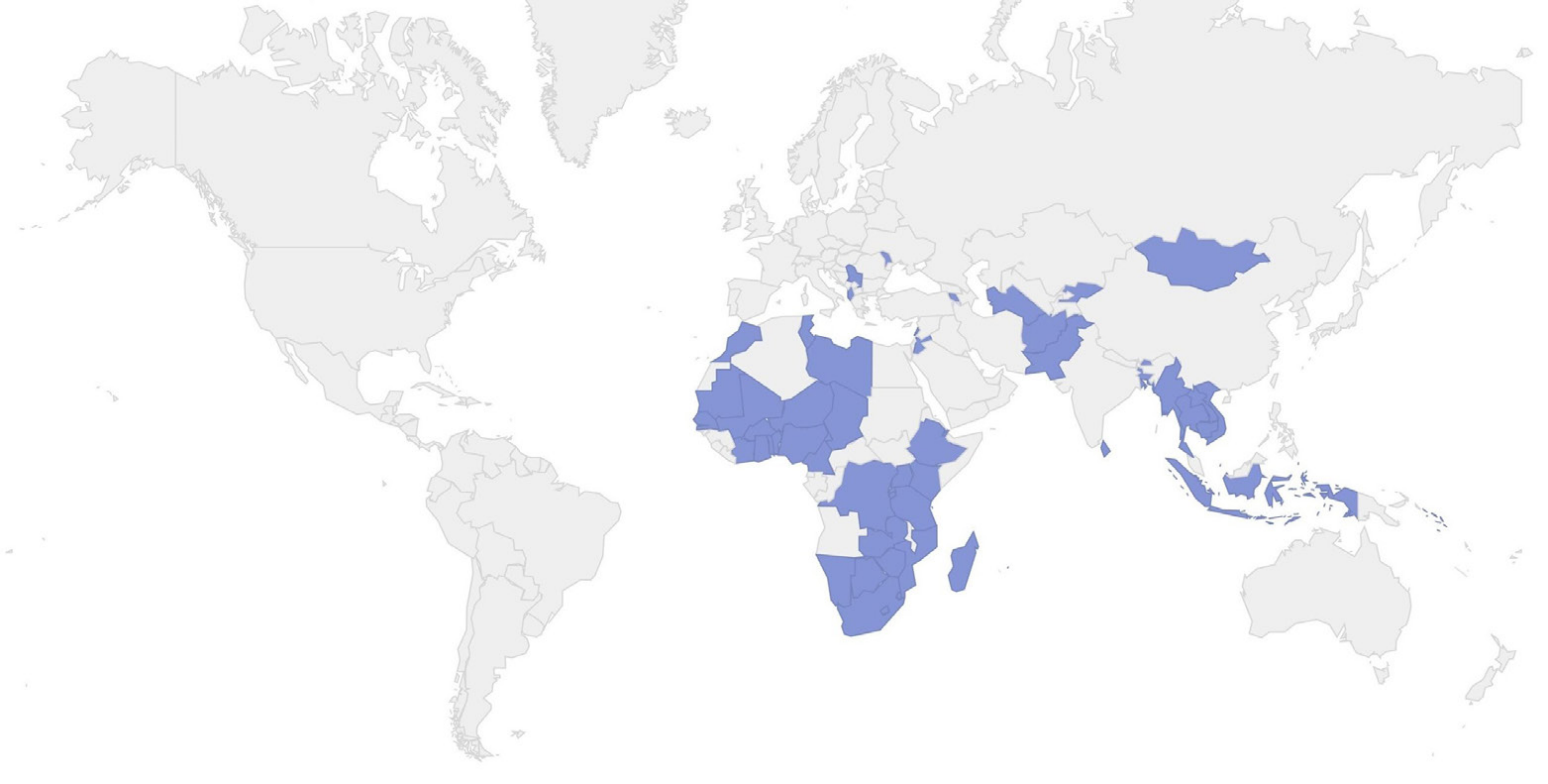
The Geographic Distribution of the 59 Countries Included in Analysis.

 All countries that had completed a JEE and had publicly available scores by November 30, 2019 were eligible for inclusion. We excluded high-income countries, as defined by the World Bank, that had completed assessments, as they were not likely to have received extensive AHS (ie, Australia, Bahrain, Belgium, Canada, Finland, Japan, Kuwait, Latvia, Lithuania, Oman, Qatar, Saudi Arabia, Seychelles, Singapore, Slovenia, South Korea, Switzerland, the United Arab Emirates, and the United States). We also excluded countries that were impacted by the West African Ebola Epidemic because it was difficult to disentangle financing provided for capacity-building from financing for outbreak response during the time period in question (ie, Guinea, Liberia, and Sierra Leone). Countries experiencing widespread social conflict or war were also excluded from our analysis because we expected that their public health capacities would likely be degraded, funding notwithstanding (ie, Central African Republic, Eritrea, South Sudan, and Sudan). Finally, countries with JEEs published in 2019 were excluded because we lacked current year funding data (ie, Iraq, Malawi, North Macedonia, and the Republic of Congo). The resulting dataset included a total of 59 countries (Figure 1).

###  Variables of Interest

 Our principal independent variable of interest was disbursed AHS funding. Our analyses scaled expenditure as a continuous variable, where a one-unit change corresponded to 1 million USD disbursed. Our dependent variable of interest was a country’s health security capacity assessment score (ie, scaled e-SPAR and JEE scores). Assessment scores for laboratory, surveillance, capacity, and emergency response capacities were considered primary outcomes because they often receive the greatest amount of support from donors and development partners. We considered assessment scores for additional health security capacities as secondary outcomes. We excluded several health security capacities including immunization, reporting, linking public health and security authorities, and medical countermeasure and personnel deployment, points of entry, chemical events, and radiological emergencies were excluded from our analysis because of insufficient AHS data.

 Our analyses also adjusted for several variables. We obtained each country’s population and gross domestic product (GDP) in the year in which the JEE was conducted from the World Bank’s Development Indicator Catalog.^20^ We treated population and GDP as continuous variables after rescaling them. Additionally, we categorized each country by WHO region (ie, AFRO, EMRO, EURO, SEARO, WPRO) and adjusted for the region using a series of indicator variables.

###  Data Analysis

 After rescaling the health security capacity assessment scores, we binned them into four levels by quartile. The resulting binned, ordinal variates were our outcomes of interest. To assess the impact of disbursed funds over time, we employed a distributed lag model (DLM). DLMs are specialized types of varying coefficient and dynamic models in which the effects of an exposure occur over time, as opposed to all at once.^21,22^ These methods have been used in pollution exposure modelling,^23^ but have an application to this context as the effects of disbursed AHS can be considered a type of lagged exposure. Accordingly, we examined lags of the concurrent year, one year, and two years. To account for the ordered nature of each outcome, we implemented our DLMs in a Bayesian ordinal regression framework.^24^ The additional advantage of the Bayesian setting was the ability to penalize the lagged effects via our prior selection. This was necessary as the lagged effects were highly correlated. Typically, a ridge regression would be employed in this context to penalize the coefficients which in turn would reduce the impact of correlation.^25^ In the Bayesian context, ridge regression corresponds to placing exchangeable Gaussian priors, centered at zero, on the coefficients corresponding to the correlated covariates.^26^ For each ordinal outcome in country i, Y_i_, the models had the following form:

 g[P(Y_i_ ≤ j)] = b_0_ + b_1_ Population_0i_ + b_2_ GDP_0i_ + b_3_ 1(Year_i_ = 2017) + b_4_ 1(Year_i_ = 2018) + b_5_ 1(Region_i_ = EMRO) + b_6_ 1(Region_i_ = EURO) + b_7_ 1(Region_i_ = SEARO) + b_8_ 1(Region_i_ = WPRO) + a_0_ Lag_0_ + a_1_ Lag_1_ + a_2_ Lag_2_,

 Where g[] is a link function, P(Y_i_ ≤ j) denotes the probability that Y_i_ is less than or equal to level j of the ordered response, and 1() denotes the indicator function.

 As suggested by the formulation, we controlled each DLM for the country-specific population and GDP in the year the JEE was completed. Further, we also controlled for the year of completion and the region. We placed flat, non-informative priors on the “b” coefficients and used the ridge penalty priors on the “a” coefficients, which correspond to the lagged capacity-specific disbursed funds. We selected the probit link function because the outcome was created by binning a pseudo-continuous variable. All model parameters were judged to have converged on 20 000 total posterior samples, after discarding the first 10 000 per standard practice,^27^ using trace plots and the Geweke convergence diagnostic.^28^ We conducted a sensitivity analysis using standard, frequentist cumulative link probit models that yielded similar results. However, due to the inability of the standard approaches to easily penalize the lagged coefficients, we used the Bayesian ordinal DLM to conduct inference. We examined all outcomes and lagged disbursed funds but considered results with a 95% posterior probability of an association—the probability the posterior is above or below zero – to be strong evidence of association.

 To conduct inference, we constructed 95% credible intervals (CI) which correspond to the middle 95% of the sampled posterior distribution for each parameter. Further, we determined the posterior probability of each model coefficient being larger than zero, denoted with p_0_. Estimated coefficients suggest changes in the probability of moving from the current category to the next. Positive coefficients suggest an increased probability while negative coefficients suggest decreased probabilities.

 To aid in interpretation, we also estimated marginal effects of changes in expenditures on those capacities that exhibited evidence of effects. Marginal effects were calculated with covariates held at their observed values and can be interpreted as the adjusted probability of improving a JEE quartile for a given increase in expenditure. Our R code and dataset are freely available online (https://github.com/markjmeyer/CGHSS).

## Results

 Nineteen of the countries included in the analysis conducted JEEs in 2016, 23 conducted JEEs in 2017, and 17 conducted JEEs in 2019 (Table 1). Of these, 31 were located in the WHO AFRO region, eight in the EMRO region, six in the EURO region, eight in the SEARO region, and six in the WPRO region. The median concurrent year population (in millions) was 12.3 (Q1: 3.0, Q3: 32.1) and the median concurrent year GDP (in billions, USD) was 18.6 (Q1: 9.3, Q3: 50.2).

**Table 1:** Summary Statistics of Country Level Covariates

**Year of JEE**	** No. (%)**
2016	19 (32.2%)
2017	23 (40.0%)
2018	17 (28.8%)
**WHO Region**	** No. (%)**
AFRO	31 (52.4%)
EMRO	8 (13.6%)
EURO	6 (10.2%)
SEARO	8 (13.6%)
WPRO	6 (10.2%)
**Population (millions)**	**Median (Q1, Q3)**
Concurrent year	12.3 (3.0, 32.1)
**GDP (billions, USD)**	**Median (Q1, Q3)**
Concurrent year	18.6 (9.3, 50.2)

Abbreviations: JEE, Joint External Evaluation; GDP, gross domestic product. Year and region are summarized with counts (percentages) while the concurrent year population and GDP are summarized with the median (Q1, Q3).

 At the 95% posterior probability level required for strong evidence, associations between funding and capacity level were observed for all time lag intervals but were most frequently observed on the one-year lag time interval (Table 2). Funding was found to have a positive effect on zoonosis in the year it was disbursed. Similarly, funding had a positive effect on legislation, laboratory, workforce, and risk communication capacities one year after it was disbursed. Funding was found to have a negative effect on laboratory and emergency response capacities two years after disbursal.

**Table 2:** Estimated Effects From Bayesian Ordinal Regression Models for Funding Disbursed in the Concurrent Year (a_0_), for Funding Disbursed 1 Year Prior (a_1_), and for Funding Disbursed 2 Years Prior (a_2_) Across All Health Security Capacities

**Capacity**	**Concurrent Year (a**_0_ **)**		**1-Year Lag (a**_1_ **)**		**2-Year Lag (a**_2_ **)**	
	**Estimate (95% CI)**	**p**_0_	**Estimate (95% CI)**	**p**_0_	**Estimate (95% CI)**	**p**_0_
Legislation	-0.011 (-0.031, 0.002)	0.061	0.029 (0.009, 0.056)	0.999	0.019 (-0.016, 0.063)	0.850
Coordination	-0.004 (-4.698, 4.371)	0.498	-0.004 (-4.244, 4.543)	0.499	-0.098 (-5.929, 3.568)	0.470
AMR	0.395 (-0.199, 1.039)	0.902	-0.128 (-0.964, 0.621)	0.373	-0.101 (-0.942, 0.726)	0.402
Food safety	-0.363 (-2.160, 0.961)	0.275	-0.074 (-1.565, 1.671)	0.453	0.512 (-0.444, 1.947)	0.838
Biosafety	-0.036 (-0.601, 0.523)	0.447	0.271 (-1.143, 1.925)	0.659	0.082 (-1.698, 1.734)	0.545
Zoonosis	0.019 (0.001, 0.045)	0.976	-0.011 (-0.041, 0.014)	0.209	-0.0262 (-0.063, 0.005)	0.056
Laboratory	-0.169 (-0.490, 0.140)	0.149	0.618 (0.055, 1.219)	0.983	-0.409 (-0.763, -0.077)	0.009
Surveillance	0.020 (-0.021, 0.063)	0.838	-0.179 (-0.474, 0.032)	0.056	0.008 (-0.417, 0.439)	0.515
Workforce	0.008 (-0.005, 0.021)	0.880	0.019 (0.000, 0.038)	0.976	-0.026 (-0.062, 0.009)	0.069
Preparedness	0.012 (-0.005, 0.036)	0.899	0.005 (-0.020, 0.029)	0.663	-0.018 (-0.042, 0.002)	0.043
Emergency response	-0.395 (-1.556, 0.148)	0.119	-0.127 (-1.655, 1.338)	0.429	-0.693 (-2.940, -0.012)	0.021
Risk communication	-0.079 (-0.220, 0.051)	0.117	0.197 (-0.003, 0.417)	0.973	-0.077 (-0.187, 0.028)	0.075
Overall	0.000 (-0.013, 0.013)	0.526	0.005 (-0.015, 0.025)	0.681	-0.003 (-0.013, 0.007)	0.258

Abbreviation: AMR, antimicrobial resistance. Estimates represent the median of the posterior samples for the corresponding coefficients and the posterior probability is the probability the distribution of the posterior samples is above 0.

**Figure 2:**
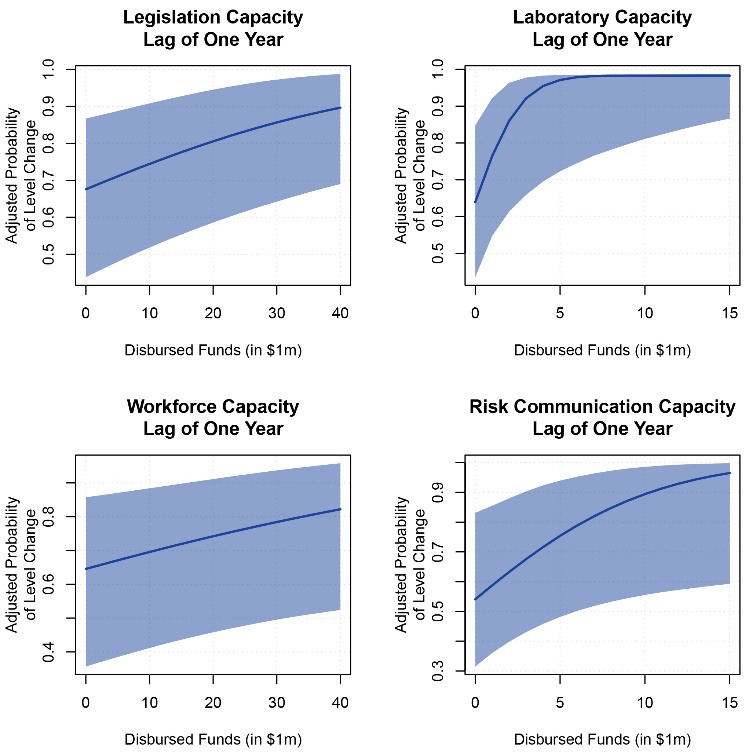
Marginal Effect of Changes in Disbursed Funds (Millions, USD) for Lags With Sufficient Evidence of Effect on Legislation, Laboratory, Workforce, and Risk Communication Capacities. Solid blue lines represent the posterior median, shaded blue bands depict the 95% pointwise credible intervals. All estimates control for population size, GDP, year, and WHO region.

 Adjusting for the region, population, GDP, and year, greater funding in the year concurrent with JEE was associated with higher JEE quartiles for zoonotic disease capacity (Table 3). We estimate that the adjusted probability of increasing one JEE quartile changes to 75.7% (95% CI: 51.7-90.9%) at the third quartile from 58.8% (95% CI: 34.5-83.1%) at the first quartile (difference of 4.2 million USD). Disbursed funding was associated with a one-level improvement in JEE quartile for legislation, laboratory capacity, workforce, and risk communication after one year (Table 3, Figure 2). Holding the same variables constant, the estimated probability of increasing a JEE quartile with disbursement at the third rather than first quartile was 74.0% (95% CI: 50.3-90.5%) versus 67.9% (95% CI: 44.2-86.9%) for legislation (difference of 890,000 USD); 67.4% (95% CI: 47.1-86.7%) versus 63.9% (95% CI: 43.5-83.7%) for laboratory capacity (difference of 26 000 USD); 69.2% (95% CI: 40.7-88.2%) versus 64.7% (95% CI: 35.9-85.8%) for workforce capacity (difference of 890,000 USD); and 69.8% (95% CI: 44.6-91.4%) versus 54.1% (95% CI: 31.5-83.1%) for risk communication capacity (difference of 353 000 USD).

**Table 3:** Adjusted Probabilities of Level Change at First Quartile (Q1), Median, and Third Quartile (Q3) of Disbursed Funds (Millions, USD)

**Capacity**	**Disbursed Funds ** **(Millions, USD)**		**Adjusted Probability of Level ** **Change (95% CI)**
Zoonotic	Q1	1.42	0.588 (0.345, 0.831)
	Median	12.90	0.640 (0.404, 0.851)
	Q3	43.10	0.757 (0.517, 0.909)
Legislation	Q1	0.39	0.679 (0.442, 0.869)
	Median	1.56	0.687 (0.452, 0.873)
	Q3	9.29	0.740 (0.513, 0.905)
Laboratory	Q1	0.00	0.639 (0.435, 0.847)
	Median	0.08	0.651 (0.447, 0.853)
	Q3	0.26	0.674 (0.471, 0.867)
Workforce	Q1	0.31	0.647 (0.359, 0.858)
	Median	2.03	0.656 (0.369, 0.863)
	Q3	9.16	0.692 (0.407, 0.882)
Risk communication	Q1	0.00	0.541 (0.315, 0.831)
	Median	0.26	0.553 (0.328, 0.837)
	Q3	3.53	0.698 (0.446, 0.914)

Reported probabilities for zoonotic capacities are for concurrent year; reported probabilities for legislation, laboratory, work force, and risk communication capacities are for one year, lagged.

## Discussion

 To our knowledge, this work represents the first published effort to quantify the impacts, over time, of AHS on health security capacity. As the COVID-19 pandemic subsides in the coming years, donors and development partners are likely to dedicate significant resources and funding to improving capacities for preventing, detecting, and responding to public health emergencies. Our results provide a foundation for a crucial evidence base elucidating where AHS is achieving its desired impact of improving capacity. They also answer a call for investigating the extent to which evaluation-derived information influences capacity development decisions.^29^

 Our results show strong evidence that AHS funding has positive effects on zoonotic disease capacities the year it is disbursed, and positive effects on legislation, laboratory, workforce, and risk communication capacities one year after it is disbursed. These associations remained, even when adjusting for region, population, GDP, and year. Future research should continue to investigate the mode by which AHS impacts these capacities and other factors that could improve or hinder the effectiveness of funding.

 Laboratory capacities present a perplexing situation from an AHS perspective. Relatively small amounts of funding were associated with substantial increases in capacity one year after disbursal. However, two years after disbursal, funding was associated with decreases in laboratory capacity. At face value, this suggests that AHS yields short term benefits for laboratory capacity, but negative effects in the longer term. However, this interpretation should be made with caution for several reasons. First, the positive one-year effects are larger than the negative two-year lag effects, so the overall effect of AHS on laboratory capacity may be interpreted as beneficial in the timeframe considered. Second, a majority of the counties included in the study did not receive funding for laboratory capacity that could be analyzed on a two-year timescale, which could render the results inaccurate. This stance is further supported by the large confidence intervals that suggest the estimate is imprecise. Future work should continue to explore the relationship between AHS and laboratory capacity as more time passes and additional data generated to more accurately determine the nature of this association.

 Perhaps of greater importance, however, is that we could not detect demonstrable positive effects of AHS on coordination, AMR, food safety, biosafety, surveillance system, or response preparedness capacities over time. This finding has enormous policy implications and is concerning, as a fundamental assumption of DAH and international assistance more broadly is that funding will yield measurable positive effects on capacity development. It is possible that this finding stems from the timeframe considered or the amount of AHS disbursed and future work should continue to investigate if these relationships change over longer periods of time or as additional funding is disbursed. It is also possible, as discussed below, that incompletely addressed endogeneity in funding decisions confounds these results. However, we hypothesize, that in addition to recognized best practices in foreign aid, such as avoiding fragmentation and distributing funding in enabling policy environments,^30^ potential predictors of success will be the extent to which developing countries set their own health security priorities and whether development partners align their efforts to these priorities. Our hypothesis stems from tenets of the Paris Declaration on Aid Effectiveness and could, for example, impact the amount of funds or other commitments a recipient country is willing to make towards matching external donor funds to support health security capacity building. Future research that captures domestic allocations for health security could also help resolve this. Irrespective of our speculation, this result underscores a compelling need for a more nuanced approach for allocating AHS and for additional work investigating current practices, how the effectiveness of AHS may be improved, and other factors impact or are predictive of health security capacity.

 It is also important to acknowledge that international AHS is not the only source of financing available for the development and strengthening of health security capacities. The 2018 revision of the JEE Tool explicitly includes financing for the implementation of IHR capacities as an indicator for the capacity relating to policy and financing.^31^ Indeed, many countries have provided domestic financing for health security that could influence assessment scores of health security capacities. While our models would have ideally captured these data, unfortunately there are currently no standards for reporting domestic health security financing, and as such, these data are not systematically captured by National Health Accounts. This represents one clear limitation in our study and a practical area for future policy action.

 The results presented have several additional limitations. Although widely used, the results of the JEE and e-SPAR are difficult to validate without large scale outbreaks or public health emergencies. Other work has demonstrated that JEE performance correlates well with baseline health, demographic and economic data, suggesting that the tool is accurately measuring core capacities.^32^ Still, it is difficult to know if these data accurately reflect the true state of health security capacities or if they are inclusive of all of the necessary considerations. For instance, several countries that received high scores in health security capacity assessments have mounted relatively poor responses to the coronavirus disease 2019 (COVID-19) pandemic (eg, the United Kingdom and United States of America) and there is a perception that JEE results have not been correlated with the effectiveness of country pandemic response.^33^ Following the COVID-19 pandemic, it seems likely that there will be an emerging body of literature on the validity of these measures and how effective they are for assessing capacities necessary for preventing, detecting, and responding to public health emergencies. For instance, discussions surrounding how poor politics and governance can undermine health security capacity are already beginning to emerge.^34^ These future efforts may generate additional frameworks that can be used for evaluating the effects of AHS on capacity development over time.

 Further, changes to outcome measurements over time precluded the analysis of capacity over time. As a result, we could investigate the association between the most recent level of capacity development and funding levels, but could not investigate year-to-year change as a function of funding. While our use of DLMs allowed us to ensure the correct time-order between funding and outcomes, we could not fully control for funding levels being higher in countries with worse expected outcomes in order to improve those outcomes. The inability to address this confounds our results and would likely bias estimates toward lower values, so our results should be interpreted underestimates of the true associations between funding and improvements in health security capacity.

 Our study also relied on AHS data contained in the Georgetown Infectious Disease Atlas’ Global Health Security Tracking Dashboard.^18^ The data contained therein relies on publicly available data and the accurate reporting of financial assistance commitments and disbursals. While a robust database, some AHS disbursals may be missing from our analysis if they were not included in this resource. Furthermore, data were dropped from our analysis, as necessitated by appropriate levels of reporting detail. This resource only presents international flows of AHS and does not capture domestic financing of health security capacities, which may also influence capacity strengthening and development. We cannot predict how these additional data would impact our results, although we strongly support the development of health security indicators in national health accounts to better allow for tracking of these data.

 Finally, the results presented in this study may not be generalizable to all countries or contexts. Several countries were excluded from our study, including those experiencing large-scale infectious disease outbreaks (prior to COVID-19) and conflict. We excluded these countries in efforts to control potential confounders, but they undoubtedly warrant additional investigation on the premise that the effects of AHS on health security capacity may exhibit unique relationships in these contexts and these areas are arguably the most in need of strong health security systems and capacities.

## Conclusion

 In conclusion, this study presents findings that provide an evidence base for informing health security capacity development decisions. Future work, including mixed-methods research, must be conducted to validate the results of this study as we will need a global commitment to health security capacity building to ensure that our world is better prepared for the epidemics and pandemics of tomorrow as we work to overcome COVID-19. These capacity building efforts and resource allocation must rely on an evidence-based to ensure funds are not wasted, capacities are sustained, and our world becomes safer.

## Ethical issues

 This work did not involve human and/or animal participants and was thus exempt from IRB review.

## Competing interests

 Authors declare that they have no competing interests.

## Authors’ contributions

 RK conceptualized the study and designed the system to track AHS funding. MRB, MJM, JDK, and RK contributed to the study design. MRB conducted data collection and transformation. MJM conducted data analysis. MRB drafted the initial manuscript. MRB, MJM, JDK, and RK contributed to the review and revisions of the manuscript, which has been approved by all of the authors.

## Funding

 The Open Philanthropy Project provided financial support to the Georgetown University Center for Global Health Science & Security that supported this work. The funding source had no role in study design, data collection, data analysis, the decision to publish, or preparation of the manuscript.
